# Ellagitannins from Raspberry (*Rubus idaeus* L.) Fruit as Natural Inhibitors of *Geotrichum candidum*

**DOI:** 10.3390/molecules21070908

**Published:** 2016-07-13

**Authors:** Elżbieta Klewicka, Michał Sójka, Robert Klewicki, Krzysztof Kołodziejczyk, Lidia Lipińska, Adriana Nowak

**Affiliations:** 1 Institute of Fermentation Technology and Microbiology, Lodz University of Technology, Wolczanska 171/173, Lodz 90-924, Poland; lidia.lipinska@dokt.p.lodz.pl (L.L.); adriana.nowak@p.lodz.pl (A.N.); 2 Institute of Food Technology and Analysis, Lodz University of Technology, Stefanowskiego 4/10, Lodz 90-924, Poland; robert.klewicki@p.lodz.pl (R.K.); krzysztof.kolodziejczyk@p.lodz.pl (K.K.)

**Keywords:** antifungal, ellagitannins, raspberry fruit, *Geotrichum candidum*

## Abstract

The paper presents the chemical characteristics of ellagitannins isolated from raspberry (*Rubus idaeus* L.) fruit and their in vitro and in situ antifungal activity against *Geotrichum candidum* ŁOCK 0511. The study investigated a complex preparation containing various raspberry ellagitannins at a concentration of 86% *w*/*w*, as well as pure lambertianin C and sanguiin H-6. The ellagitannin preparation was obtained by extracting raspberry press cake and purifying the extract using Amberlite XAD resin, while individual compounds were isolated by means of preparative HPLC. The complex preparation was analyzed for the content of ellagitannins, anthocyanins, and flavan-3-ols using HPLC and LC-MS. The antifungal activity of the complex ellagitannin preparation and the isolated ellagitannins was determined for the strain *Geotrichum candidum*. The MIC and MFC values (10.0 mg/mL and 30.0 mg/mL, respectively) were found to be the same for lambertianin C, sanguiin H-6, and the complex ellagitannin preparation. The fungistatic activity of the studied ellagitannin preparation at a concentration of 10 mg/mL, as determined by the poisoned medium method, was 65.2% following 6 day incubation of *Geotrichum candidum*, with the linear growth rate of only 16.2 mm/day. The corresponding parameters for the control sample were 0% and 56 mm/day, respectively. The study demonstrated both in vitro and in situ antifungal activity of raspberry ellagitannins against *Geotrichum candidum*.

## 1. Introduction

Ellagitannins constitute a complex class of polyphenols characterized by one or more hexahydroxydiphenoyl (HHDP) moieties esterified with a sugar, usually glucose. The HHDP group is biosynthetically formed through intramolecular, oxidative (C-C) coupling of suitably oriented neighboring galloyl residues in galloylglucoses. Ellagitannins exhibit very high structural variability due to the different ways in which HHDP residues can be linked with the glucose moiety, and especially due to their strong tendency to form dimeric and oligomeric forms [1,2]. High ellagitannin content has been reported for strawberries, cranberries, cloudberries, and red raspberries. The main raspberry ellagitannins are sanguiin H-6 (1871 g/mol) and lambertianin C (2805 g/mol). Both compounds are dimers and trimers of casuarictin (936 g/mol) respectively, which are formed by intermolecular C-O bonds between an HHDP group and a galloyl group on a neighboring monomer [1]. The content of these compounds in fresh raspberry fruit, depending on the cultivar, ranges from 360 to 750 mg/kg for sanguiin H-6 and from 280 to 630 mg/kg for lambertianin C [3]. Polyphenols, and especially ellagitannins and flavonoids, have a considerable antimicrobial potential. Many studies have shown that extracts from pomegranate and other plants rich in ellagitannins exhibit a significant inhibitory effect on *Escherichia coli*, *Salmonella enteritidis*, *Staphylococcus aureus*, *Bacillus subtilis*, *Klebsiella pneumoniae*, *Pseudomonas aeruginosa*, *Yersinia enterocolitica*, *Listeria monocytogenes*, and *Candida albicans*, as well as on molds such as *Alternaria alternata*, *Fusarium oxysporum*, and *Rhizoctonia solani* [4,5,6,7].

*Geotrichum candidum* has been isolated from a variety of environments, including river water, air, food, and animal feed [8]. Being used in the food industry, this fungus has been subjected to numerous biotechnological studies in that area. While in the past *G. candidum* was usually found safe, with a low pathogenic potential, recently it has been reported to cause an increasing number of fungal infections. *G. candidum* is an opportunistic microorganism [9]. If present in food, this species may infect cancer patients treated with chemotherapy, HIV-positive individuals, those subjected to long-term antibiotic therapy or using corticosteroids, as well as diabetics [9,10]. Furthermore, the problem may be expected to further intensify with the increasing population of patients with immune deficiencies. Therefore, it seems necessary to seek safe antifungal compounds or preparations that could be used as natural preservatives in the food industry. The study presents the chemical characteristics of ellagitannins isolated from raspberry (*Rubus idaeus* L.) fruit and their in vitro and in situ antifungal activity against *Geotrichum candidum*.

## 2. Results and Discussion

### 2.1. Ellagitannin Identification in REP by LC-MS

Six ellagitannins were identified in the obtained raspberry extract (Table 1) in the process of gradient elution with polyphenols being purified on XAD 1600 resin. Due to the protocol applied, the extract was free of, amongst others, low molecular weight ellagitannins, ellagic acid and its conjugates, and other polyphenols, such as some anthocyanins and flavan-3-ols. Compounds with retention time of 14.57 min and 17.72 min gave a major [M − H]^−^ ion at *m*/*z* 1567.15 and a doubly charged ion at *m*/*z* 783.07. Fragmentation of the doubly charged ion gave a sequence of singly charged ions characteristic of sanguiin H-10 isomers. However, it should be noted that the first isomer fragmented to *m*/*z* 1235, which was associated with the loss of an HHDP (hexahydroxydiphenic acid) residue as described by Kähkönen et al. [11], while the second isomer fragmented to *m*/*z* 1265, which was linked to the loss of a galloylated glucose as described by Gasperotti et al. [3]. Despite the same mass, the two compounds have different structures, as reported by Gasperotti et al. [3].

The compound with a retention time of 15.13 was characterized by the presence of a doubly charged ion at *m*/*z* 1250, which fragmented to an ion at *m*/*z* 2200 losing an HHDP residue as well as to an ion at *m*/*z* 1867 losing a galloylated glucose. Based on the obtained fragmentation and literature data [3], this compound was identified as lambertianin C without one ellagic acid residue. Compounds with retention times of 19.25 and 19.75 exhibited the presence of a doubly charged ion at *m*/*z* 1401, which fragmented to *m*/*z* 1869, 1567, and 1235, indicating the presence of lambertianin C.

The occurrence of two forms suggests the presence of isomers of this compound, which were also observed by Hager et al. [12] and McDougall et al. [13]. In our case, the first compound was identified as an isomer of lambertianin C and the other one as the main (also in quantitative terms) lambertianin C found in raspberry fruit. The compound with a retention time of 20.57 min gave an ion at *m*/*z* 1869, which characteristically fragmented to *m*/*z* 1567, 1235, 935, 633, and 301, indicating the presence of sanguiin H-6. The identification of lambertianin C and sanguiin H-6 was also confirmed by comparing retention times and MS spectra of the isolated standards. The same fragmentation patterns have been reported by Gasperotti et al. [3], Hager et al. [12], and Mertz et al. [14].

### 2.2. Quantitative Composition of Phenolic Compounds in REP

**Figure 1:**
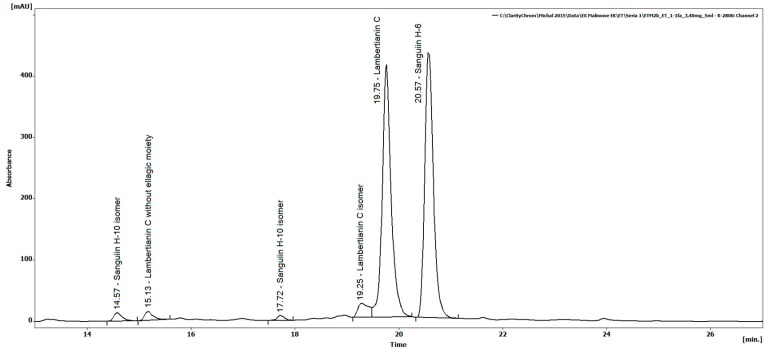
HPLC chromatogram of the raspberry ellagitannin preparation (REP) with UV detection at 250 nm.

Table 2 shows the quantitative ellagitannin composition of REP. In general, ellagitannins account for 92% of total phenolics. The other compounds, that is, flavan-3-ols and anthocyanins constitute 7.4% and 0.9% of total phenolics, respectively. The most abundant ellagitannins in REP are lambertianin C and sanguiin H-6, which collectively account for 93% of total ellagitannins (Figure 1), with the remaining ones being sanguiin H-10 isomers and lambertianin C derivatives. Ellagic acid, a product of ellagitannin hydrolysis, was not detected. The results show that the applied method of extraction (with 60% acetone) and purification (with XAD 1600 resin) leads to a product characterized by a high phenolic content, with more than 86% being hydrolysable tannins, including ellagitannins.

### 2.3. Inhibition of Geotrichum Candidum Growth by Ellagitannins

The strain *Geotrichum candidum* ŁOCK 0511 used in the presented study, had been isolated as a contaminant of non-pasteurized milk. In the ellagitannin antagonism test, this strain exhibited an inhibition zone of 10.3 ± 0.47 mm for REP and of 9.3 ± 0.47 mm and 8.3 ± 1.69 mm for LC and SH6, respectively. The MIC and MFC for REP were 10.0 mg/mL and 30.0 mg/mL, respectively, and were identical as those for the LC and SH6 fractions. REP, which is a complex preparation containing both lambertianin C (44.156 g/100 g) and sanguiin H-6 (35.917 g/100 g) (Table 3) exhibits similar antagonistic activity to the pure compounds isolated in this study (Table 3). In respect of chemical structure, sanguin H-6 is a dimer, while lamberitanin C is a trimer of casuarictin. Hence, taking into consideration their molar masses, in the MIC analysis we introduce the same mass but different amount of moles for both compounds. At the same time, the number of functional groups involved in interactions remains the same. Our research has not identified any link between the degree of polymerization or synergism of both compounds and the MIC parameter. This resulted in the same MIC parameter for the REP preparation, which included 80% of these compounds in total. It should also be noted that REP preparation also contained 7% of the flavanols, which in terms of the MIC parameter may compensate for lower ellagitannin content. Foss et al. [15] observed a similar effect, they concluded that crude extract from pomegranate and isolated compound punicalagin showed about the same value of MIC. In this case, punicalagin was the main substance of the crude extract. Moreover, application of the REP preparation in food or pharmaceutical industry for production of an extract rich in ellagitannins would be technologically easier and significantly less expensive than isolation of pure compounds. Therefore, in further study we used REP rather than individual ellagitannin fractions.

The antifungal activity of extracts from *Rubia tinctorum*, *Carthamus tinctorius* and *Juglans regia* against *Geotrichum candidum* was studied by Mehrabian et al. [16], who reported effective inhibition of fungal growth by aqueous and methanol extracts from *Carthamus tinctorius* (40 mm and 20 mm, respectively). However, Mehrabian et al. [16] did not specify the chemical characteristics of the plant extracts. In turn, Talibi et al. [17] studied the antagonistic activity of 43 preparations obtained from 36 plant species against *Geotrichum candidum*. One of the tested substances was a preparation from the leaves and shoots of *Rubus ulmifolius* Schott, with the MIC and MFC values of 0.3125 mg/mL and >5 mg/mL, respectively. Foss et al. [15] studied antifungal activity of crude extract and punicalagin from pomegranate against *Trichophyton* sp. and *Microsporium* sp. which are human dermatophytes. The authors determine the MIC values of crude extract of pomegranate for these dermatophytes are respectively 125 μg/mL and 250 μg/mL. Research conducted by Webster et al. [18] specified the antagonistic activity of water-based plant extracts (*Heracleum maximum*, *Aralia nudicaulis*, *Glycyrrhiza lepidota*, *Fragaria virginiana*, *Populus tremuloides*, *Artemisia frigida*, *Artemisia campestris*, *Solidago gigantea*, *Alnus viridis*, *Potentilla simplex*, *Juniperus communis*, *Epilobium angustifolium*, *Betula alleghaniensis*, *Acorus calamus*) against 23 human fungal pathogens belonging to the genera: *Candida*, *Saccharomyces*, *Cryptococcus*, *Trichophyton*, *Microsporum*, *Epidermophyton*, *Aspergillus*, *Fusarium*, *Rhizopus*. Researchers proved that the antifungal activity of plant extracts is individuated and it depends on the extract and the test strains. For instance, for the *Fragaria virginiana* extract, the MIC parameters were from 400mg/L in case of *Trichophyton mentagrophytes*, *Microsporum canis*, *Epidermophyton floccosum*, *Aspergillus fumigatus* to 50000 mg/L for *Aspergillus flavus*, *Fusarium saloni* strains. In the analysis provided by Thirach et al. [19], the antifungal activity of an ethanol extract of *Acorus calamus* was assessed against 28 clinical isolates of *Candida* sp. using a broth microdilution technique. The MIC was identified as 28,800 ± 16,320 mg/L, and *Acorus calamus* was interpreted in this setting as being a potential fungistatic agent.

### 2.4. The Fungistatic Activity (FA) of Ellagitannins and the Linear Growth Rate (T) of Filamentous Fungi in Their Presence

The fungistatic activity (FA) of ellagitannins (REP) and the linear growth rate (T) of the strain *Geotrichum candidum* ŁOCK 0511 in their presence was determined (Table 4). In the experiments, REP concentrations were selected based on prior studies concerning MIC and MFC.

The FA of the raspberry ellagitannin preparation against *G. candidum* increased with ellagitannin concentration in the growth medium. A REP concentration of 10 mg/mL led to FA of 66.4% after 24 h of incubation, which was maintained on day 6. An ellagitannin concentration lower than MIC (1.0 mg/mL) resulted in FA of 24.7% after 24 h of incubation and 13.8% on day 6. In the study of Tabei et al. [17], the FA of a preparation from *Rubus ulmifolius* Schott at a concentration of 5 mg/mL was 15%. The studied preparation (from *Rubus idaeus* L.) at a concentration of 5 mg/mL exhibited FA ranging from 49.4% to 54.5%, depending on incubation time (Table 4). The linear growth rate (T) for *Geotrichum candidum* ŁOCK 0511 ranged from 51.9 mm/day for an ellagitannin concentration of 1.0 mg/mL to 16.2 mm/day for 10.0 mg/mL. In contrast, in the control samples T was from 56.0 mm/day to 61.5 mm/day (Table 4). The T values obtained for *G. candidum* indicate effective fungal inhibition and are statistically significant with respect to the control sample.

### 2.5. In Situ Effect of Ellagitannin Preparation

**Figure 2:**
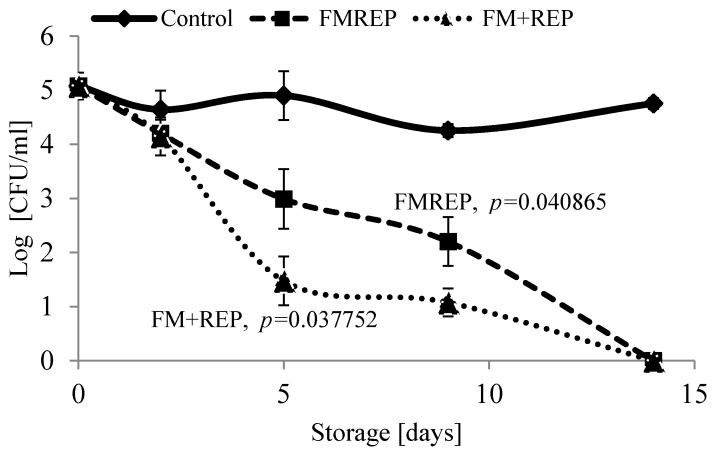
Effect of ellagitannins (10 mg/mL REP) on *Geotrichum candidum* viability in fermented milk during refrigerated storage (4 °C). FMREP: milk fermented with ellagitannins, FM + REP: ellagitannins added after completed fermentation.

Hydrolysable tannins are known to effectively inhibit the growth of Gram-negative bacteria, such as *Salmonella* sp., *E. coli*, *Helicobacter pylori*, *Campylobacter* sp. and Gram-positive bacteria: *Staphylococcus* sp., *Clostridium* sp., and *Bacillus* sp. [20]. In contrast, they do not inhibit Gram-positive bacteria, such as *Lactobacillus* sp., or do so only to a limited extent [21,22,23]. This gives the opportunity to use ellagitannin preparations in functional foods, especially those containing probiotics. This fact was at the core of the design of in situ tests for the inhibition of a food-contaminating fungus. The selected matrix was milk fermented by the probiotic strain *Lactobacillus acidophilus* ŁOCK 0842. The contaminant, *Geotrichum candidum*, was a pleomorphic filamentous fungus, often termed yeast-like [24]. Experiments were conducted in two variants: the ellagitannin preparation (REP) was added either prior to lactic fermentation (FMREP) or after it (FM + REP). During storage, the control exhibited the presence of *Geotrichum candidum* ŁOCK 0511 at a level of 5 log units (LU) throughout the storage period (Figure 2). In the FMREP sample, *G. candidum* counts decreased by 2 LU (to approx. 3 LU) on day 5 of storage and no live cells of this fungus were detected on day 15. In the FM + REP sample, *G. candidum* counts declined to 1.5 LU on day 5 of storage and no live fungal cells were detected on day 15, similarly to the FMREP sample.

Both samples supplemented with ellagitannins (FMREP and FM + REP) exhibited the same effect, that is, the absence of live *G. candidum* cells in 1 mL of fermented milk after 15 days of refrigerated storage. Nevertheless, in the FM + REP sample, to which the ellagitannin preparation was added following fermentation, *G. candidum* counts were lower by 1.5 LU than in the FMREP sample as early as on day 5 of storage. In the case of FMREP, the ellagitannin preparation was added before the process of milk fermentation. *Lactobacills acidophilus* bacteria were involved in the milk fermentation process, which may have provoked partial hydrolysis of the compounds. Osawa et al. [25] showed the presence of tannase and gallate decarboxylase in strains of *Lactobacillus* species isolated from humam feces and fermented food. Ellagitannins under the influence of tannase are hydrolyzed into hexahydroxydiphenic acid, which later on is developed into ellagic acid [26]. Thus, changes in the formula of ellagitannins which may occur during the milk fermentation are likely to influence the dynamics of the antagonistic activity against *G. candidum* in the FMREP test between the 5th and 10th day of the process.

### 2.6. Evaluation of Macroscopic and Microscopic Morphology of Geotrichum Candidum ŁOCK 0511

**Figure 3:**
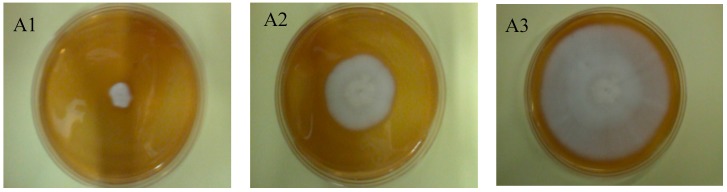

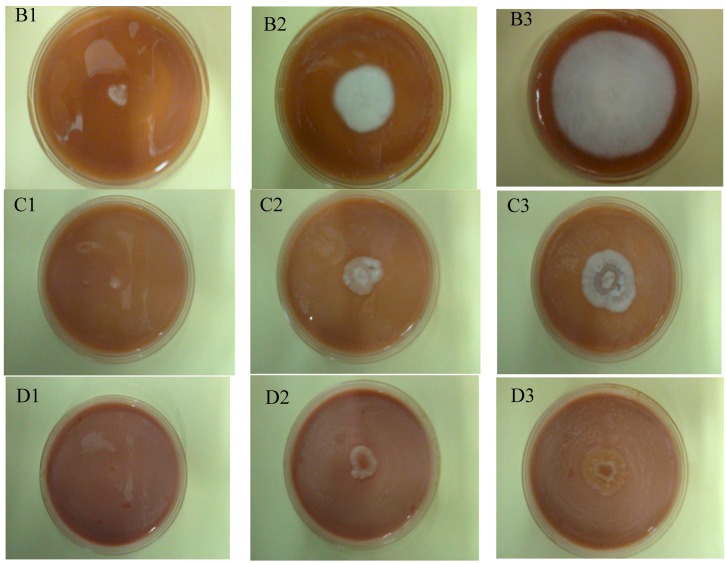
*Geotrichum candidum* growth on a solid medium supplemented with ellagitannins (REP) (**A**) Control: 1: after 1 day of incubation, 2: after 3 days of incubation, 3: after 6 days of incubation; (**B**) 1.0 mg/mL REP: 1: after 1 day of incubation, 2: after 3 days of incubation, 3: after 6 days of incubation; (**C**) 5.0 mg/mL REP: 1: after 1 day of incubation, 2: after 3 days of incubation, 3: after 6 days of incubation; (**D**) 10.0 mg/mL REP: 1: after 1 day of incubation, 2: after 3 days of incubation, 3: after 6 days of incubation.

Macroscopically, the growth of *G. candidum* in the presence of 1.0 mg/mL REP in the medium did not differ from that of the control sample (Figure 3B). In both cases, the colonies were white and velvety. Differences appeared upon the supplementation of the medium with 5.0 mg/mL and 10 mg/mL of REP. At 5 mg/mL REP in the medium, the fungal colonies were still white, but lost their smooth and velvety surface structure; they became wavy with a spherical pattern. The growth of *G. candidum* in the presence 10 mg/mL REP (Figure 3D) was significantly different from that in the control sample. The fungal colonies were yellowish without a velvety luster and had a wavy surface.

**Figure 4:**
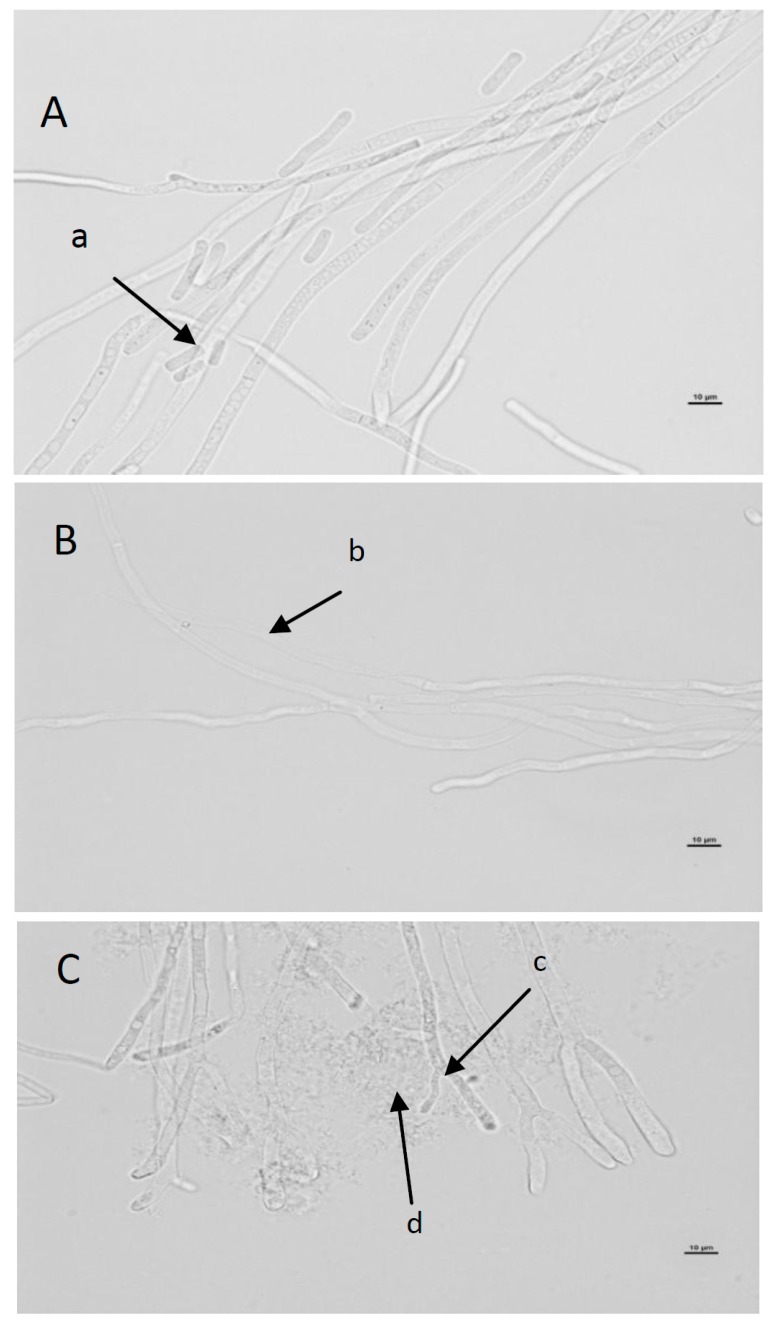
Morphology of the hyphae of *Geotrichum candidum* cultured in the presence of ellagitannins (REP) on day 6. (**A**) control, (**B**) 5. 0 mg/mL REP, (**C**) 10.0 mg/mL REP. a: arthrospores, b: dehydrated hyphae, c: forked tips, d: hyphal lysis. Scale bar is 10 μm.

Microscopic slides with living *G. candidum* cells cultured in the presence of 5 mg/mL and 10 mg/mL REP were made (see Figure 3). In the control sample, the hyphae were straight with rounded tips, septa were visible, and arthrospores were present (Figure 4A-a). The microscopic slide prepared from *G. candidum* cultured on a medium containing 5.0 mg/mL REP did not reveal arthrospores, and the hyphae were contorted with rounded tips. Furthermore, the hyphae were deformed due to dehydration (Figure 4B-b). Finally, microscopic examination of the *G. candidum* culture supplemented with 10.0 mg/mL REP revealed substantial in the morphology of the fungus. The hyphal tips were forked (Figure 4C-c), in contrast to the controls. Cell lysis was observed and no arthrospores were detected. As can be seen, ellagitannin preparation obtained from red raspberry fruit prevented spore formation in *G. candidum* ŁOCK 0511 and altered hyphal morphology leading to cell wall disorganization and leakage of cytoplasmic material from the hyphae.

Similar results were reported by Gatto et al. [27], who studied the antifungal activity of extracts from polyphenol-rich wild herbs against fungi causing fruit and vegetable spoilage: *B. cinerea* (grey mold), *M. laxa* (inducing brown rot of stone fruits) *P. digitatum* (Pers.) Sacc. (green mold affecting citrus fruits); *P. italicum* Wehmer (blue mold affecting citrus fruits), *P. expansum* Link. (blue mold affecting apples), *A. carbonarius* (Bainier) Thom, and *A. niger*. Gatto et al. [27] also observed changes in hyphal morphology, inhibition of spore formation and cell lysis.

This behavior suggests progressive poisoning of the germ tube caused by some “toxic” compounds in the extract. The greater effect of the ellagitannin extracts on the germ tube elongation rather than on arthrospore germination is probably due to the different composition of the cell wall and to its need to absorb nourishing substances from outside, including also the “toxicants” found in the extract [28]. It can be assumed that the light lipophilic nature of some phenolic compounds contributes to their progressive accumulation in the cell membrane of the fungal pathogens, thus altering its permeability and affecting some transport mechanisms [27,29].

## 3. Materials and Methods

### 3.1. Plant Material

Frozen raspberry fruits of the Polka cultivar collected in August 2013 from plantations in Lublin Province were purchased from the company Cajdex (Łódź, Poland). The fruits were stored in polyethylene bags at −18 °C prior to ellagitannin extraction.

### 3.2. Fruit Processing and Ellagitannin Extraction

Ellagitannins were extracted from raspberry press cake, following juice pressing from fruit pulp. Prior to processing, 30 kg of fruits was defrosted and ground using a fruit grinder (Model 886.9, Zelmer, Rzeszów, Poland). The pulp was heated to 50 °C and treated with the enzymatic preparation Rohapect 10 L (AB Enzymes, Darmstadt, Germany) at a dose of 0.2 mL per 1 kg of pulp. Enzyme treatment lasted for 1 h at 50 °C, with the pulp stirred every 10 min. Subsequently, the pulp was pressed using a laboratory basket press (self-manufactured, Lodz University of Technology, Lodz, Poland) to separate juice from the press cake. The latter was extracted with water (1/2 of press cake weight) for 15 min at 50 °C to remove water-soluble anthocyanins. Next, the press cake was pressed again to obtain secondary juice and press cake (3.4 kg).

The press cake was extracted in two steps using 60% acetone at ambient temperature. The mass ratio of the press cake to the extractant was 1:5 and extraction time was 8 h per one step. The extraction process was augmented with shaking using an orbital shaker (Elmi DOS-10 L, Aizkraukles, Riga, Latvia) at 150 rpm. After the first step, raw extract was filtered through cotton filter cloth in order to separate solids, while in the second step, the post-extraction residue was extracted according to the same procedure. The extracts obtained from the two steps were mixed and filtered through a 3.6 mm thick Hobrafilt S40N cellulose filter with 5 µm nominal retention (Hobra-Školnik S.R.O., Broumov, Czech). Acetone was removed from the raw extract using a Basis Hei-VAP HL rotary vacuum evaporator (Heidolph, Schwabach, Germany) at 60 °C and under reduced pressure of 450–72 mbar. The extract without acetone was again passed through a Hobrafilt S40N filter, and then purified on a 90 cm × 1.6 cm column packed with Amberlite XAD 1600 N resin (DOW, Midland, MI, USA). The extract was injected at a rate of approx. 15 mL/min. Elution was carried out at 10 mL/min using water/ethanol solutions with ethanol concentrations of 10%, 20%, 30%, 40%, 50%, and 60%, consecutively. The volume of solutions with each ethanol concentration equaled the volume of the column. The eluate obtained using the 40% ethanol solution contained the highest concentrations of lambertianin C and sanguiin H-6. Subsequently, ethanol was removed and the eluate was concentrated to approx. 5° Brix using a Basis HEI-VAP HL rotary vacuum evaporator at 60 °C under pressure reduced from 135 to 72 mbar. The concentrated extract was then freeze-dried (−32 °C, 48 h; Christ, Alpha 1–2 LDplus, Osterode am Harz, Germany). In the process, 30.8 g of raspberry ellagitannin preparation (REP) was obtained in the form of a red powder.

### 3.3. Isolation of Sanguiin H-6 and Lambertianin C

Individual ellagitannins were isolated from an aqueous REP solution with a concentration of 4 mg/mL using a Knauer preparative HPLC system (Berlin, Germany). The chromatograph was composed of two gradient-forming pumps (Knauer K-501), a Luna 10 u C18 (2) 100A AXIA-packed (250 mm × 21.2 mm; 10 μm) column (Phenomenex, Torrance, CA, USA), a UV-Vis detector, a Foxy R1 Teledyne ISCO fraction collector (Lincoln, NE, USA), and Eurochrom 2000 chromatographic software. Two eluents were used for separation: eluent A—0.1% formic acid in water, eluent B—75% methanol. The flow rate was 15 mL/min. The following gradient was used: 0–5 min 10% B; 5–30 min 10%–25% B; 30–50 min 25%–35% B; 50–65 min 35%–40% B; 65–70 min 40%–10% B; and 70–75 min 10% B. The injection volume was 500 μL. The detection parameter was set to 260 nm. Lambertianin C and sanguiin H-6 peaks were collected from 200 separations. Methanol was removed from the obtained solutions using a Basis HEI-VAP HL rotary vacuum evaporator (Heidolph, Schwabach, Germany) at 60 °C under a pressure of 100 mbar. Then, the solutions were freeze-dried, producing 96.5 mg of sanguiin H-6 (white-beige powder) and 102 mg of lambertianin C (white-creamy powder).

### 3.4. Identification of Ellagitannins

A Dionex Ultimate 3000 high performance liquid chromatograph (HPLC) from Thermo Fisher Scientific (Germering, Germany) coupled with a diode array detector (DAD,) and a Q Exactive Orbitrap mass spectrometer (MS, Thermo Fisher Scientific, Bremen, Germany) were used for the identification of ellagitannins. The solvents used for separations were as follows: solvent A, 1% (*v*/*v*) formic acid in water and solvent B, an 80:20 (*v*/*v*) acetonitrile:water solution. The following gradient was used: 0–6.5 min 5% (*v*/*v*) B; 6.5–12.5 min 5%–15% (*v*/*v*) B; 12.5–44 min 15–45% (*v*/*v*) B; 44–45 min 45%–75% (*v*/*v*) B; 45–50 min 75% (*v*/*v*) B; 50–52 min 75%–5% (*v*/*v*) B; 52–65 min 5% (*v*/*v*) B. A Luna C18 (2) 100A 250 × 4.6 mm i.d., 5 μm column was used with a Luna C18 4 mm × 3 mm pre-column (both from Phenomenex). The column temperature was set to 35 °C, the flow rate was 1 mL/min, and the injection volume was 20 μL. Chromatographic data were collected using Xcalibur software (Thermo Fisher Scientific, Waltham, MA, USA). The MS system coupled to the HPLC apparatus was an Orbitrap mass spectrometer equipped with an H-ESI probe used in the negative mode. The source parameters were as follows: vaporizer temperature 500 °C, ion spray voltage 4 kV, capillary temperature 400 °C, with sheath gas and auxiliary gas flow rates being 75 and 20 units, respectively. The detector was operated in either the full MS or full MS/dd-MS^2^ scan modes. In the full MS mode, the scan rage of *m*/*z* 200–2000 was used. To generate MS^2^ data, the full MS/dd-MS^2^ scan mode was used. In this mode, the selected precursor ions entered into an HDC collision cell, where they were fragmented with normalized collision energy (NCE) to obtain product ion spectra (MS^2^). In our experiments, the NCE used to generate MS^2^ spectra was set to 20. Tuning and optimization were performed using direct injection of a REP diluted in an 80:20 (*v*/*v*) mixture of mobile phases A and B at a flow rate of 0.25 mL/min. The results of ellagitannin identification are given in Table 2.

### 3.5. Quantification of Ellagitannins

First, 3 mg of REP was dissolved in 5 mL 50% methanol and the solution was sonicated for 5 min. Subsequently, the solution was diluted with mobile phase A at a ratio of 1:1 (*v*/*v*), centrifuged at 15,000× *g* (MPW-260R centrifuge; Med. Instruments, Warsaw, Poland) and transferred into a vial. The content of ellagitannins was determined using a Smartline chromatograph (Knauer, Berlin Germany), composed of a degasser (Manager 5000), two pumps (P1000), an autosampler (3950), a thermostat, and a PDA detector (2800). Ellagitannins were separated on a 250 mm × 4.6 mm i.d., 5 µm Gemini C18 110A column (Phenomenex, Torrance, CA, USA) by gradient elution with 0.05% (*v*/*v*) phosphoric acid:water (solvent A) and 83:17 (*v*/*v*) acetonitrile:water with 0.05% phosphoric acid (solvent B). The column temperature was set to 35 °C, the flow rate was 1.25 mL/min, and the gradient program was as follows: 0–5 min 5% (*v*/*v*) B; 5–10 min 5%–15% (*v*/*v*) B; 10–35 min 15%–40% (*v*/*v*) B; 35–40 min 40%–73% (*v*/*v*) B; 40–44 min 73% (*v*/*v*) B; 44–46 min 73%–5% (*v*/*v*) B; 46–54 min, 5% (*v*/*v*) B. The injection volume was 20 μL. Data were collected using ClarityChrom v. 3.0.5.505 software (Knauer). Ellagitannins were detected at 250 nm, and standard curves for lambertianin C and sanguiin H-6 were used for quantification. The sanguiin H-6 curve was used for the quantification of sanguiin H-10 isomers. The lambertianin C curve was used for the quantification of lambertianin C derivatives.

### 3.6. HPLC Analysis of Anthocyanins

Anthocyanins in REP were quantified pursuant to the protocol given in Sójka et al. [30]. The same apparatus and separation conditions were used. A standard curve using an external standard of cyanidin-3-*O*-glucoside was used.

### 3.7. HPLC Analysis of Flavanols (Sum of proAnthocyanidins and Catechins)

Flavanols were quantified in REP by acid-catalyzed degradation of polymeric proanthocyanidins in excess phloroglucinol, as described in Sójka et al. [31]. The same apparatus and separation conditions were used. Prior to analysis, samples of fruits, juices, and press cake were freeze dried for 48 h at −36 °C (Christ, Alpha 1-2 LDplus, Osterode am Harz, Germany).

### 3.8. Microorganisms

The study involved *Geotrichum candidum* 0511, a strain of filamentous fungi deposited with the Collection of Industrial Microorganisms of the Institute of Fermentation Technology and Microbiology ŁOCK 105, Lodz University of Technology (Poland). Two morphotypes of *Geotrichum candidum* have been described in the literature. The first one forms cream-colored yeast-like colonies which produce abundant arthrospores, but grow poorly. The other morphotype forms white felt-like colonies similar to those of molds, consisting of vegetative septate hyphae with few arthrospores, and is capable of fast and abundant growth [9,10]. The strain *Geotrichum candidum* ŁOCK 0511, which was used in this study, belongs to the latter morphotype.

The strain was stored on YEA slants (Yeast Extract Agar, Sigma-Aldrich, Saint Louis, MO, USA) at 4 °C and activated either on Sabouraud dextrose broth or on agar (Merck KGaA, Darmstadt, Germany), as need be.

### 3.9. Antagonistic Activity of Ellagitannins

Antagonistic activity was assessed by the standard disk diffusion method. Sterile paper discs (5.0 mm diameter, Oxoid, Hampshire, UK) were impregnated with 20 μL of ellagitannin preparation (REP), lambertianin C (LC) and Sanguiin H-6 (SH6) at a concentration of 30 mg/mL, and placed on Sabouraud dextrose agar (Merck) plates inoculated with 100 μL of fungal suspension adjusted to 10^6^ colony forming units (CFU) per mL. Discs containing 50 mg/mL dimethyl sulfoxide (DMSO, Merck) were used as a negative control. Experiments were done in triplicate and the results were presented as mean inhibition of strain growth (after subtracting disc diameter) ± standard deviation. One-way analysis of variance (ANOVA) with the Bonferroni *post hoc* test (*p* ≤ 0.05) was applied to determine differences between REP, LC and SH6 samples.

### 3.10. Inhibition of Geotrichum Candidum Growth by Ellagitannins

The antifungal properties, minimal inhibitory concentration (MIC), and minimal fungicidal concentration (MFC) of the ellagitannin preparation (REP), lambertianin C (LC) and Sanguiin H-6 (SH6) were determined by the broth dilution method in test tubes. Ellagitannin solutions were prepared and diluted in DMSO (50 mg/mL) (Merck). Subsequently, 100 μL of diluted ellagitannin solutions were added to 4.9 mL of Sabouraud dextrose broth (Merck) containing a suspension of arthrospores at a concentration of 10^5^ spores/mL, so that the final ellagitannin concentration ranged from 0.05 mg/mL to 30 mg/mL. The samples were incubated at 30 °C for 72 h. The minimum inhibitory concentration was defined as the lowest concentration of the compound that inhibited visible growth of the fungus. Subsequently, 1 mL was collected from the samples with no visible growth and plated on Sabouraud dextrose agar. The samples were incubated at 30 °C for 48 h. The minimum fungicidal concentration was defined as the lowest concentration of the compound leading to no fungal growth [32].

### 3.11. Fungistatic Activity and Linear Growth Rate of Filamentous Fungi in the Presence of Ellagitannins

Ellagitannin-induced inhibition of the linear growth rate of *Geotrichum candidum* ŁOCK 0511 was studied by means of the poisoned medium method [33]. Sabouraud dextrose agar (Merck) was supplemented with 1.0 mg/mL, 5.0 mg/mL, and 10.0 mg/mL of REP (the amounts specified refer to ellagitannin content). The control was a culture without REP. Tests were done in triplicate. The linear growth rate of *Geotrichum candidum* (T) was calculated based on fungal growth measurements performed every 24 h according to the formula:

T = (A/D) + (b_1_/d_1_) + … + (b_n_/d_n_) (mm/24 h) (1)

where T—linear growth rate, A—mean fungal colony diameter in mm, D—duration of experiment, b_1_…b_n_—colony diameter growth in mm, d_1_…d_n_—number of days since the last measurement.

The fungistatic activity of ellagitannins (FA) against filamentous fungi was determined based on fungal growth inhibition calculated according to the formula:

FA = ((K − A)/K) × 100 (%)
(2)

where: FA—fungistatic activity in %, K—mean fungal colony diameter on the control plate, A—mean fungal colony diameter on the plate containing ellagitannins. The experimental data are expressed as mean values. One-way analysis of variance (ANOVA, *p* ≤ 0.05) was applied to find differences between the experimental samples and the control samples.

### 3.12. In Situ Effect of the Raspberry Ellagitannin Preparation

The in situ antagonistic activity of REP against *Geotrichum candidum* was determined in fermented milk. Reconstructed 10% milk (skimmed powdered milk, from the company BTL sp. z o.o., Lodz, Poland) was pasteurized at 80 °C for 10 min. After cooling down, the milk was inoculated with the *Lactobacillus acidophilus* ŁOCK 0842 strain of lactic acid bacteria (Collection of Industrial Microorganisms of the Institute of Fermentation Technology and Microbiology ŁOCK 105, Lodz University of Technology) in the amount of 1.5 × 10^6^ CFU/mL and supplemented with an aqueous solution of the ellagitannin preparation (REP), for ellagitannin concentration in milk to reach 10 mg/mL. Milk prepared in this way (FMREP) was incubated for 48 h at 37 °C. Alternatively, in some samples REP was added to milk following fermentation, with the other parameters remaining constant (FM + REP).

Milk samples (FMREP and FM + REP) were inoculated with *Geotrichum candidum* in the amount of 1.2 × 10^5^ CFU/mL. The obtained fermented milk, inoculated with the fungus, was stored under refrigeration (4 °C). During storage, *G. candidum* counts were monitored by the plate method on days 2, 5, 9, and 15. The control sample was fermented milk inoculated with the fungus following lactic fermentation, without REP.

Experiments were conducted in triplicate with the results given as mean values with standard deviations. One-way analysis of variance (ANOVA, *p* ≤ 0.05) was applied to find differences between samples with REP and the control samples.

### 3.13. Microscopic Analysis

Photomicrographs of *Geotrichum candidum* mycelium were acquired with a Nikon Eclipse Ci H600L (Nikon, Tokyo, Japan) microscope (total magnification 400×) operated with NIS-Elements Advanced Research v. 3.0 software (Nikon).

## 4. Conclusions

Given the increasing numbers of immunologically compromised patients, it is of utmost importance to control microbiological contaminants in food. The widely used chemical preservatives may give rise to allergic disorders and are not very well accepted by the consumers. Therefore, it is necessary to seek natural biologically active compounds which could be applied as food bio-preservatives. The present study demonstrated that ellagitannins obtained from red raspberry (*Rubus idaeus* L.) fruit exhibit in vitro and in situ antifungal activity against *Geotrichum candidum*.

## Figures and Tables

**Table 1:** LC-MS identification of ellagitannins in the raspberry ellagitannin preparation (REP).

R_t_ (min)	MS Data	MS/MS Value	MS/MS Data	Tentative Structural Assignment
14.57	[1567.15]^−1^ [783.07]^−2^	783	[1235.07]^−1^ [935.08]^−1^ [633.07]^−1^ [469.01]^−1^ [301.00]^−1^	Sanguiin H-10 isomer
15.13	[1250.60]^−2^ [833.40]^−3^	1250	[2200.19]^−1^ [1867.14]^−1^ [1567.14]^−1^ [1235.07]^−1^ [933.06]^−2^ [633.07]^−1^ [301.00]^−1^	Lambertianin C without ellagic moiety
17.72	[1567.15]^−1^ [783.07]^−2^	783	[1265.14]^−1^ [1103.09]^−1^ [935.08]^−1^ [933.07]^−1^ [633.07]^−1^ [469.01]^−1^ [301.00]^−1^	Sanguiin H-10 isomer
19.25	[1401.01]^−2^ [934.07]^−3^	1401	[1869.14]^−1^ [1567.14]^−1^ [1235.07]^−1^ [935.08]^−1^ [633.07]^−1^ [301.00]^−1^	Lambertianin C isomer
19.75	[1401.01]^−2^ [934.07]^−3^	1401	[1869.14]^−1^ [1567.14]^−1^ [1235.07]^−1^ [935.08]^−1^ [633.07]^−1^ [301.00]^−1^	Lambertianin C (standard)
20.57	[934.07]^−2^ [1869.14]^−1^	934	[1567.14]^−1^ [1235.07]^−1^ [935.08]^−1^ [633.07]^−1^ [301.00]^−1^	Sanguiin H-6 (standard)

**Table 2:** Ellagitannin (REP), anthocyanin (ACY), and flavanol (FLAVA) content in raspberry ellagitannin extract (REP).

Compound	Mean (mg/100 g)	SD
**Ellagitannins**		
Sanguiin H-10 isomer 1	1065	57
Lambertianin C without ellagic moiety	1456	97
Sanguiin H-10 isomer 2	731	47
Lambertianin C isomer	2961	332
Lambertianin C	44156	1705
Sanguiin H-6	35917	1300
Total ellagitannins	86287	3537
**Other Phenolics**		
Total anthocyanins	823	6
Total flavanols (mDP)	7003 (1.9)	158 (0.1)

Results are given as means of three replicates ± standard deviation (SD); mDP: mean degree of proanthocyanidin polymerization.

**Table 3:** Minimal inhibitory concentration (MIC), minimal fungicidal concentration (MFC), and antagonistic activity of lambertianin C (LC), sanguiin H-6 (SH6), and the raspberry ellagitannin preparation (REP) with respect to *Geotrichum candidum* LOCK 0511.

Test	REP	LC	SH6
MIC (mg/mL)	10.0	10.0	10.0
MFC (mg/mL)	30.0	30.0	30.0
Zone of growth inhibition (mm) ± SD	10.3 ± 0.47 ^a^	9.3± 0.47 ^a^	8.3± 1.69 ^a^

SD: standard deviation, ^a^ no statistically significant differences were found in analysis of variance (ANOVA) with the Bonferroni *post hoc* test (*p* ≥ 0.05).

**Table 4:** Fungistatic activity (FA) of the raspberry ellagitannin preparation (REP) and the linear growth rate (T) for *Geotrichum candidum*.

Ellagitannins (mg/mL)	FA (%)				Growth Rate T_Ellagitannins_ (mm/day)	Growth Rate T_Control_ (mm/day)
	Time (days)					
	1	2	3	6		
1.0	24.7	27.5	16.7	13.8	51.9 *	61.5
5.0	54.5	49.4	50.3	51.4	33.9 **	56.0
10.0	66.4	60.1	74.4	65.2	16.2 ***	56.0

* statistically significant difference T_Ellagitannins_ versus T_Control_ (*p* = 1.28 × 10^−6^). ** statistically significant difference T_Ellagitannins_ versus T_Control_ (*p* = 3.09 × 10^−4^). *** statistically significant difference T_Ellagitannins_ versus T_Control_ (*p* = 1.49 × 10^−7^).
